# High Prevalence of Vitamin B12 Deficiency and No Folate Deficiency in Young Children in Nepal

**DOI:** 10.3390/nu9010072

**Published:** 2017-01-17

**Authors:** Bernadette N. Ng’eno, Cria G. Perrine, Ralph D. Whitehead, Giri Raj Subedi, Saba Mebrahtu, Pradiumna Dahal, Maria Elena D. Jefferds

**Affiliations:** 1 Epidemic Intelligence Service, Division of Scientific Education and Professional Development, Centers for Disease Control and Prevention, 1600 Clifton Road, Atlanta, GA 30333, USA; uyt0@cdc.gov; 2 Division of Nutrition, Physical Activity & Obesity, Centers for Disease Control and Prevention, 4770, Buford Hwy, Chamblee, GA 30341, USA; hgk3@cdc.gov (C.G.P.); rnw2@cdc.gov (R.D.W.J.); 3 Child Health Division, Ministry of Health and Population, Kathmandu 44600, Nepal; subedi.giriraj@gmail.com; 4 Nutrition Section, UNICEF, UN House, Pulchowk, Lalitpur, P.O. Box 1187, Kathmandu 44600, Nepal; smebrahtu@outlook.com (S.M.); pdahal@unicef.org (P.D.)

**Keywords:** serum B12, red blood cell folate, animal source foods

## Abstract

Many children in low- and middle-income countries may have inadequate intake of vitamin B12 and folate; data confirming these inadequacies are limited. We used biochemical, demographic, behavioral and anthropometric data to describe the folate and vitamin B12 concentrations among six- to 23-month-old Nepalese children. Vitamin B12 (serum B12 < 150 pmol/L) and folate deficiencies (red blood cell (RBC) folate < 226.5 nmol/L) were assessed. We used logistic regression to identify predictors of vitamin B12 deficiency. The vitamin B12 geometric mean was 186 pmol/L; 30.2% of children were deficient. The mean RBC folate concentration was 13,612 nmol/L; there was no deficiency. Factors associated with vitamin B12 deficiency included: (a) age six to 11 months (adjusted odds ratio (aOR) 1.51; 95% confidence interval (CI): 1.18, 1.92) or 12–17 months (aOR 1.38; 95% CI: 1.10, 1.72) compared to 18–23 months; (b) being stunted (aOR 1.24; 95% CI: 1.03, 1.50) compared to not being stunted; (c) and not eating animal-source foods (aOR 1.85; 95% CI: 1.42, 2.41) compared to eating animal-source foods the previous day. There was a high prevalence of vitamin B12 deficiency, but no folate deficiency. Improving early feeding practices, including the consumption of rich sources of vitamin B12, such as animal-source foods and fortified foods, may help decrease deficiency.

## 1. Introduction

Folate and vitamin B12 are essential micronutrients which are critical especially during infancy and early childhood as these are periods of rapid growth, development, and increased demand. During infancy and childhood, deficiency of folate and vitamin B12 can result in megaloblastic anemia, poor growth, and increased infections [1,2,3,4]; additionally, deficiency of vitamin B12 can potentially cause irreversible neurologic damage to the developing brain [4,5]. Vitamin B12 is exclusively found in animal-derived foods such as meat, eggs, fish and milk, and its deficiency is mainly due to inadequate dietary intake of these foods [6], except where foods are fortified. A mother’s strict vegetarian diet may be associated with vitamin B12 deficiency both in the mother and in the newborn, because of the increased demand during pregnancy and lactation [7]. Concentrations of vitamin B12 in breast milk reflect maternal vitamin B12 stores [7,8], and maternal vitamin B12 stores are often depleted among women in low-income countries, including up to one-third of women in rural Nepal [9,10]. Plasma and serum concentrations of vitamin B12 are a relatively good indicator of body stores [11]. Folate deficiency mainly results from a low intake of green leafy vegetables, legumes and meat [12]. Folate deficiency is presumably less prevalent compared to vitamin B12 deficiency among young children because of its abundance in breast milk independent of maternal stores and intake, and the large-scale fortification of flour and grains with folic acid in many low-income countries [8,13,14].

According to the Nepal Demographic and Health Survey of 2011, undernutrition remains a public health problem as stunting, underweight and wasting affect 49%, 39% and 13% of children less than five years of age, respectively [15]. This suggests micronutrient deficiencies are likely a problem in Nepal, but limited population-based micronutrient status data exist for Nepali children. Varying deficiency levels have been described in some specific sub-populations. A recent population-based study in the Bhaktapur municipality of Nepal identified 17% vitamin B12 deficiency among breastfed two- to 12-month-old infants [16]. Another study among six- to 35-month-old Nepalese children presenting to a clinic with diarrhea found a vitamin B12 deficiency of 41% [17]. Poor micronutrient intake including vitamin B12 and folate has also been described among lactating women in Bhaktapur and is partly attributable to poor dietary diversity and a low intake of micronutrient-rich foods [18]. Also, studies of pregnant women in some districts of Nepal showed that the prevalence of vitamin B12 deficiency was common, with 49% in Kathmandu [9] and 28% in the Sarlahi district [10]. Despite the importance of addressing child malnutrition, there is a dearth of population-based biochemical data assessing the micronutrient status among children in Nepal. The purpose of this analysis is to describe folate and vitamin B12 status among a representative sample of children aged six to 23 months in the Achham and Kapilvastu districts of Nepal and to identify independent predictors of deficiency.

## 2. Materials and Methods

### 2.1. Survey Design and Sampling 

A cross-sectional household cluster survey was conducted in Achham and Kapilvastu districts of Nepal to collect nutritional and infant and young child feeding (IYCF) practices data for children six to 23 months of age prior to the implementation of an integrated IYCF, micronutrient powder and early child development intervention. Using population proportion to size (PPS) sampling, 40 clusters were selected in each district, for a total of 80 clusters. A household census was conducted in each cluster to identify all eligible children; 32 children in Achham and 34 in Kapilvastu were randomly selected in each cluster and 2640 eligible children were invited to participate in the survey. There was no replacement if there were fewer than the targeted number of eligible children in the cluster. Participation in the survey was voluntary and informed consent, which was witnessed and formally recorded, was obtained from each mother. The mothers were the respondent to a questionnaire and venous blood samples were collected from children. The information collected included sociodemographic characteristics, IYCF practices and foods and beverages consumed by the child the previous day. The Nepal Health Research Council gave ethical and technical approval for the survey. A de-identified data set was used for this secondary analysis. 

### 2.2. Anthropometric Measurements

The child’s recumbent length was measured lying down using a standard length-measuring board (Shorr board). Weight was measured using a lightweight electronic SECA digital scale (UNICEF Electronic Scale or Uniscale, SECA, Chino, CA, USA), which allows for weighing very young children through an automatic mother-child adjustment, that eliminates the mother’s weight while she is standing on the scale with her child. 

### 2.3. Calculating IYCF Indicators and Groups of Foods Eaten

We calculated two World Health organization (WHO) IYCF indicators [19] using maternal recall of the child’s food intake the previous day; (1) minimum meal frequency was met if a child consumed the minimum number of required meals the previous day: two or more times per day for a breastfed child aged six to eight months, three or more times for a breastfed child aged nine to 23 months and four or more times for a non-breastfed child aged six to 23 months; (2) minimum dietary diversity was met if a child consumed food from at least four of seven food groups in the previous day. The seven food groups included grains/roots/tubers; legumes and nuts; dairy products; flesh foods; eggs; vitamin A–rich fruits and vegetables; and other fruits and vegetables [19]. To assess the child’s consumption of animal-source foods in the previous day, mothers were asked if the child had breastfed; eaten flesh foods (chicken, mutton, beef, fish, poultry, liver, kidney, heart and other organ meats or blood-based food); eggs; or dairy products (milk, curd, cheese or other milk products, ghee). 

### 2.4. Blood Sampling and Biochemical Analysis

Venous blood samples were collected at the household and processed in a field laboratory the same day. Hemoglobin was measured in the field using a HemoCue™ 301 photometer (HemoCue, Angelhom, Sweden). The serum was stored in silicone coated tubes and whole blood hemolysate was stored in potassium ethanediaminetetraacetic acid (K_2_EDTA) tubes. These samples were transferred and stored frozen in the district public health centers until the end of data collection. They were then transferred and stored at −86 °C at the National Public Health Laboratory (NPHL) in Kathmandu until they were shipped to international laboratories for biochemical analyses. Serum vitamin B12 concentrations were measured using atomic absorption spectroscopy at the Jordan University of Science and Technology (JUST) Laboratory in Irbid, Jordan. Folate levels were estimated by measuring folate concentrations in red blood cell lysate using the microbiological assay at the Peking University, Institute of Reproductive and Child Health Laboratory in Beijing, China. Both the JUST and Peking University laboratories participate in the U.S. Centers for Disease Control and Prevention’s (CDC) external laboratory quality assurance program (VITAL-EQA) [20]. Both laboratories received satisfactory results for their participation in the VITAL-EQA program; JUST Laboratory received >80% precision of the VITAL-EQA results, with 8.6% bias for serum vitamin B12, and Peking University received >90% precision of the VITAL-EQA results, with 3.6% bias for folate.

### 2.5. Analytic Sample

A total of 2640 children six to 23 months old were selected and invited to participate. Of these, 2549 mothers of children completed the interviews, yielding a response rate for completing the questionnaire of 96.6%. Of the 2549 children whose mothers/caregivers completed the questionnaire interview, 82 (3.2%) were excluded from this analysis: 47 (1.8%) because mothers declined to have their children participate in blood sample collection and 35 (1.4%) because blood draw for laboratory testing was not successful. Overall, blood was successfully drawn from 2467 (93.4%) of the children invited for interview, of whom 2405 (91.1%) were tested for RBC folate and 2166 (82.0%) for serum vitamin B12; these sample sizes vary because some samples did not have sufficient volume for all biomarker assessments.

### 2.6. Statistical Analysis

We converted length and weight data to Z-scores by using World Health Organization reference values [21]. We defined stunting as length-for-age Z-score (LAZ) of less than two standard deviations and wasting as weight-for-length Z-score (WLZ) of less than two standard deviations [21]. We excluded Z-score values for six children because they were not biologically plausible; LAZ of >3 or <−5 and WLZ of >5 or <−4 [21]. Hemoglobin concentrations were adjusted for altitude following WHO [22] and anemia was defined as hemoglobin <11.0 g/dL. The RBC folate cut off for defining folate deficiency was <226.5 nmol/L, which is the cut off based on macrocytic anemia. Serum vitamin B12 deficiency was defined as <150 pmol/L [23,24]. Serum vitamin B12 data were positively skewed, so we calculated geometric means and 95% confidence intervals (CIs), and described the prevalence of vitamin B12 deficiency. Mothers were asked about their child, education, household income and household assets which were used to generate a household wealth variable. Analyses are presented by child’s age (six to 11, 12–17, 18–23 months), sex (male vs. female), mother’s education level (no education, primary level, secondary level and higher), household wealth (lowest, middle and highest), child’s nutritional status (stunted vs. non-stunted and wasted vs. non-wasted), adequate minimum dietary diversity (yes vs. no), adequate minimum meal frequency (yes vs. no) and consumption of animal-source foods (yes vs. no) in the previous day. We used Chi-square analyses to test for differences in vitamin B12 deficiency by the above groups; *p* < 0.05 was considered statistically significant. The association between the significant factors in bivariate analyses were further included in a logistic regression model. Child sex was close to significant and was also included in the model because of the known preference for boys in South Asia [25]. Variables in the final model included age, sex, mother’s education, wealth, wasting, stunting, adequate minimum dietary diversity, adequate minimum meal frequency and consumption of animal-source foods. Because the RBC folate data were normally distributed, we reported the overall mean and 95% CI. Data are not presented by socio-demographic and dietary factors for folate since no deficiency was found. Estimates are weighted to account for district population, and analyses account for complex survey design. We used SAS 9.3 (SAS Institute Inc., Cary, NC, USA) to analyze the data. 

## 3. Results

Among the 2166 children assessed for vitamin B12, 53.0% were female (Table 1). Approximately half of the mothers had no education. Wasting was prevalent in 11.6% of children, and stunting in 43.0%. Overall, 92% of children were breastfed in the previous 24 h (data not shown in the tables). Only 24.7% of children had received the recommended minimum dietary diversity and 61.8% the recommended minimum meal frequency in the previous day. Overall, 979 (45.2%) children had eaten animal-source foods in the previous day. The mean and standard deviation for hemoglobin was 11.04 ± 1.17 (data not shown in the tables); the prevalence of anemia was 44.5% (95% CI: 41.8, 47.2) (Table 1). The geometric mean for the serum B12 concentration was 186 pmol/L (95% CI: 177.9, 195.1); and 30.2% of children were vitamin B12 deficient (Table 1). In bivariate analyses, vitamin B12 deficiency varied significantly by multiple indicators as listed in Table 1, including but not limited to: wasting (38.0% vs. 29.2%), stunting (32.6% vs. 29.6%) and lack of intake of animal-source foods in the previous day (37.4% vs. 21.0%) (Table 1). 

All significant factors as well as child sex, which was close to significant in the chi-square analysis, were included in the logistic regression model. In this adjusted analysis, being younger, stunted, and not eating animal-source foods the previous day significantly predicted vitamin B12 deficiency (Table 2). The mean RBC folate was 1362 nmol/L (95% CI: 1317, 1406) and no children were folate deficient. 

## 4. Discussion

Vitamin B12 deficiency is prevalent among children six to 23 months old in two districts of Nepal, with a higher prevalence of deficiency among children aged six to 11 and 12 to 17 months. It may also be contributing to the high prevalence of anemia found in these children, although there were no significant differences in vitamin B12 deficiency by anemia status. A higher prevalence of vitamin B12 deficiency has been reported among breastfed six- 30-month-old Indian children (36% deficiency defined as vitamin B12 < 150 pmol/L) [26] and among one- to six-year-old Mexican children (36% deficiency defined as vitamin B12 < 203 pg/mL (equivalent to 150 pmol/L)) [27]. In addition, various prevalences of vitamin B12 deficiency have also been reported in population-based studies in other countries, depending on the population studied and cut-offs used to define deficiency; among the breastfed six- to 30-month-old Indian children, the prevalence of vitamin B12 deficiency increased to 48% when the cut-off for vitamin B12 was raised to <200 pmol/L, while the prevalence of vitamin B12 deficiency (<148 pmol/L) was 40% among school-aged children in rural Kenya [28]. These results suggest that vitamin B12 deficiency may be common among children in developing countries. 

The main source of vitamin B12 is animal-derived foods; therefore, the finding that not eating animal-source foods the previous day was associated with vitamin B12 deficiency was not surprising. As few population-based studies have assessed vitamin B12 status among young children, these confirmatory findings may be useful for populations with similar dietary practices. Animal-source foods might not be consumed for various reasons, including expense or cultural and religious reasons [6]. In Nepal, the staple diet, particularly for young children, is mostly vegetarian and may not include frequent intake of meat or meat products [15,29]. In our survey, we found that less than half of children had eaten animal-source foods the previous day. This suggests that child feeding practices may contribute to the low vitamin B12 concentrations. The low serum concentrations of vitamin B12 found in our study may also be partially due to low vitamin B12 body reserves among their mothers due to maternal deficiency. In developing countries, deficiency of vitamin B12 is common during pregnancy [7] and low serum B12 concentrations have also been reported in pregnant women from developed countries [14,30]. When the vitamin B12 status of women is poor during pregnancy and lactation, their infants may have smaller stores of the vitamin at birth [31] and the concentration of the vitamin in breast milk is likely to be low [7,32,33]. In our study, the majority of the children had breastfed, and it is possible that the quality of breastmilk in terms of vitamin B12 content was poor. No data on vitamin B12 status were available for the mothers of the children in our sample; however, other studies in Nepal have reported high levels of serum B12 deficiency (28%–49%) among lactating women [9,10]. Given the known adverse health effects of vitamin B12 deficiency, including poor growth, increased infection, anemia and irreversible brain damage [1,4,5], these findings might have important public health implications in the Nepal context. 

Our finding of a higher prevalence of vitamin B12 deficiency among younger children may suggest inadequate intake during the weaning period as children are transitioning from exclusive breastfeeding to breastfeeding and complementary feeding. In the multivariable analysis, the minimum meal frequency and minimum dietary diversity were not significant predictors of B12 deficiency. Potentially, this is because these variables are closely related to the consumption of animal-source foods. Our finding that children suffering from stunting were at higher odds of vitamin B12 deficiency corroborates previous studies in Nepal [34] and other developing countries [35]. Overall, our findings highlight the need to strengthen the implementation of the WHO guidelines of appropriate IYCF practices to address undernutrition in children [19,36].

We did not observe folate deficiency in our analysis, similar to other studies that have shown no or low deficiency, including no deficiency among two- to 12-month-old infants in Nepal [16]; 2% deficiency among six- to 35-month-old children presenting to the clinic with diarrhea in Nepal [17]; 3.2% deficiency among one- to six-year-old Mexican children in a nationally representative population-based study [27]; and 6% deficiency among breastfed Indian children aged six to 30 months [26]. The lack of folate deficiency in our findings may be partly explained by the high prevalence of breastfeeding (92%) among these children. The folate concentration in breast milk is generally high and to a large extent is independent of the folate status of the mother [37]. Also, there is widespread consumption of legumes in Nepal, as well as mandatory fortification with folic acid of industrially produced flour in Nepal, which may be another source for some children [14]. 

Strengths of this analysis include providing one of the few population-based estimates of vitamin B12 and folate status among children six to 23 months of age. Also, we analyzed RBC folate using the microbiological assay, which is the gold standard. Our study had several limitations: (a) the presented data were representative of children in two districts of Nepal and may not reflect the micronutrient status among young children in other parts of the country; (b) the child dietary intake information was based on mother interviews which are subject to several biases, including but not limited to recall and social desirability bias; (c) we did not collect information on maternal vitamin supplementation intake or maternal vitamin B12 status which can influence the vitamin B12 concentrations available in breastmilk; and (d) we did not assess homocysteine and methylmalonic acid concentrations, which are considered more reliable indicators of B12 deficiency than the concentration of B12 in blood.

## 5. Conclusions

This analysis revealed a high prevalence of vitamin B12 deficiency among young children aged six to 23 months in two districts in Nepal. Deficiency was associated with being younger, having stunting, and not consuming animal-source foods the previous day. We did not find folate deficiency among children. Based on these findings, it is likely that vitamin B12 deficiency may represent other underlying public health problems in Nepal that should be explored. Nationally representative data collected from young children and women of reproductive age would help to better understand the magnitude of deficiency in the country and to inform the design of programs to support appropriate infant and child feeding practices. 

## Figures and Tables

**Table 1:** Serum vitamin B12 concentrations among children aged six to 23 months by socio-demographic and nutrition variables, Kapilvastu and Accham districts, Nepal, 2012.

Characteristics	Serum Vitamin B12 Concentrations			B12 Deficient (<150 pmol/L)		
	*n*	Geometric Mean (pmol/L)	95% CI ^a^	%	95% CI	*p*-Value
	(Unweighted)					
Total	2166	186	178, 195	30.2	26.6, 33.9	
Age (in months)						
6–11	680	177	167, 188	34.2	29.9, 38.6	<0.001
12–17	845	185	176, 195	31.1	26.6, 35.6	
18–23	641	199	189, 211	24.7	20.3, 29.3	
Sex						
Female	1009	181	171, 192	32.3	27.9, 36.7	0.069
Male	1157	191	182, 201	28.5	24.5, 32.4	
Mother’s Education level						
None	1072	179	169, 188	33.5	29.0, 38.0	<0.001
Primary level	608	182	171, 194	30.5	25.6, 35.3	
Secondary and higher	486	210	200, 219	23.1	19.0, 27.2	
Wealth						
Lowest	860	177	167, 187	33.4	28.5, 38.3	0.028
Middle	445	186	173, 201	31.9	25.9, 38.0	
Highest	861	198	188, 209	26.3	22.0, 30.5	
Child wasted ^b^						
Yes	250	175	160, 190	38	30.8, 45.0	0.008
No	1913	188	180, 197	29.2	25.6, 32.7	
Child stunted ^c^						
Yes	930	179	170, 190	32.6	28.0, 37.3	0.05
No	1233	192	183, 201	29.6	24.9, 32.2	
Adequate minimum dietary diversity ^d^						
Yes	535	215	204, 226	19.6	15.5, 23.9	<0.001
No	1631	178	169, 187	33.6	29.5, 37.7	
Adequate minimum meal frequency ^e^						
Yes	1339	194	185, 204	27	23.5, 30.6	<0.001
No	827	176	166, 186	35.1	30.1, 40.1	
Intake of animal-source food ^f^						
Yes	979	209	199, 211	21	17.5, 24.5	<0.0001
No	1187	171	162, 180	37.4	32.8, 41.9	
Anemia (Hb < 11.0 g/dL) present						
Yes	963	183	174, 196	31.2	26.2, 35.8	0.068
No	1203	191	182, 201	28.9	24.7, 33.1	

^a^ 95% Confidence Intervals; ^b^ Weight-for-length Z-score <−2 standard deviations (less than −2SD) from the median of a reference population (WHO 1995). Excludes three children whose Z-scores were biologically not possible; ^c^ Length-for-age Z-score <−2 standard deviations (less than −2SD) from the median of a reference population (WHO 1995). Excludes three children whose Z-scores were biologically not possible; ^d^ Defined as food intake from at least four of the seven main food groups in the previous day. The seven food groups include grains/roots/tubers; legumes and nuts; dairy products; flesh foods; eggs; vitamin A–rich fruits and vegetables; and other fruits and vegetables (WHO 2010); ^e^ Defined as intake of the minimum number of required meals the previous day: two or more times per day for a breastfed child aged six to eight months, three or more times for a breastfed child aged nine to 23 months and four or more times for non-breastfed children aged six to 23 months (WHO 2010); ^f^ Defined as reported intake of animal flesh foods, eggs and dairy products intake in the previous day.

**Table 2:** Odds of vitamin B12 deficiency among children six to 23 months by sociodemographic, nutritional and clinical characteristics, Kapilvastu and Accham districts, Nepal 2012.

Characteristics	Adjusted Odds Ratio	95% CI ^a^
Age (in months)		
6–11	1.51	1.18, 1.92
12–17	1.38	1.10, 1.72
18–23	REF ^b^	
Sex		
Females	1.22	1.00, 1.48
Male	REF	
Mothers’ Education		
No education	1.19	0.89, 1.57
Primary level	1.15	0.86, 1.55
Secondary and higher	REF	
Wealth		
Lowest	1.01	0.77, 1.34
Middle	1.07	0.77, 1.49
Highest	REF	
Child Wasted ^c^		
Yes	1.26	0.96, 1.66
No	REF	
Child Stunted ^d^		
Yes	1.24	1.03, 1.51
No	REF	
Adequate minimum dietary diversity ^e^		
Yes	REF	
No	1.24	0.90, 1.70
Adequate minimum meal frequency ^f^		
Yes	REF	
No	1.24	0.99, 1.54
Intake of animal-source foods ^g^		
No	1.85	1.42, 2.41
Yes	REF	

^a^ 95% Confidence Intervals; ^b^ Reference ^c^ Weight-for-Length Z-score <−2 standard deviations (less than −2SD) from the median of a reference population (WHO 1995); ^d^ Length-for-age Z-score <−2 standard deviations (less than −2SD) from the median of a reference population (WHO 1995); ^e^ Defined as food intake from at least four of the seven main food groups in the previous day. The seven food groups include grains/roots/tubers; legumes and nuts; dairy products; flesh foods; eggs; vitamin A–rich fruits and vegetables; and other fruits and vegetables (WHO 2010); ^f^ Defined as intake of the minimum number of required meals the previous day: two or more times per day for a breastfed child aged six to eight months, three or more times for a breastfed child aged nine to 23 months and four or more times for non-breastfed children aged six to 23 months (WHO 2010); ^g^ Defined as reported intake of animal flesh foods, eggs and dairy products intake in the previous day.
